# Wavelet compression of off-axis digital holograms using real/imaginary and amplitude/phase parts

**DOI:** 10.1038/s41598-019-44119-0

**Published:** 2019-05-17

**Authors:** P. A. Cheremkhin, E. A. Kurbatova

**Affiliations:** National Research Nuclear University MEPhI (Moscow Engineering Physics Institute), Moscow, 115409 Russia

**Keywords:** Optics and photonics, Information technology

## Abstract

Compression of digital holograms allows one to store, transmit, and reconstruct large sets of holographic data. There are many digital image compression methods, and usually wavelets are used for this task. However, many significant specialties exist for compression of digital holograms. As a result, it is preferential to use a set of methods that includes filtering, scalar and vector quantization, wavelet processing, etc. These methods in conjunction allow one to achieve an acceptable quality of reconstructed images and significant compression ratios. In this paper, wavelet compression of amplitude/phase and real/imaginary parts of the Fourier spectrum of filtered off-axis digital holograms is compared. The combination of frequency filtering, compression of the obtained spectral components, and extra compression of the wavelet decomposition coefficients by threshold processing and quantization is analyzed. Computer-generated and experimentally recorded digital holograms are compressed. The quality of the obtained reconstructed images is estimated. The results demonstrate the possibility of compression ratios of 380 using real/imaginary parts. Amplitude/phase compression allows ratios that are a factor of 2–4 lower for obtaining similar quality of reconstructed objects.

## Introduction

Digital holography is a technique that allows two-dimensional (2D) and three-dimensional (3D) imaging of objects^1–3^. This is obtained by registering the interference pattern formed by the object and reference beams on a digital camera’s photosensor (CCD, CMOS, Foveon X3, etc.). The registered frame is a digital hologram. A 3D image of the object or 2D scenes of the registered environment can be reconstructed numerically (by model propagation of the wave using a computer)^4^ or optically (by displaying this hologram on spatial light modulator and its illumination)^5,6^. Digital holography is widely used for microobjects and particle tracking^7^, detection of changes of refractive or reflective indices^8^, measuring temperature^9^, etc. The increase in the number of pixels in digital cameras has led to the growth of the file size of holograms. The additional frame rate increase has led to considerable growth of the file size of holographic videos. Therefore, compression of holograms is an important and useful task. The different methods of compressing holographic image or video files^10–13^ can be divided into three main groups based on the following:

- image and video compression standards (JPEG, HEVC, etc.)^13–20^, including additional HEVC-coding optimization^18^ and neural network post-processing^19^;
- vector and scalar methods of quantization (iterative or noniterative)^10,21–29^, including phase-difference-based implementation^28^ and local and global thresholding binarization^29^; and
- wavelet transforms^11,30–41^, including the cosine transform^32,33^ and the Fresnelet transform^34^.
  However, several other compression methods can be considered separately from the above-mentioned groups: for example, scanning methods^33,42^ and generative approaches^43^. A number of methods can be assigned to different groups of techniques also. As an additional compression technique, lossless coding can be applied to holographic data^22,24,44–46^ (e.g., Huffman^47^, Lempel–Ziv–Welch coding^48^, and others).
  In some papers in the 1970 s, the first attempts for dividing the amplitude/phase (A/P) parts of a hologram’s Fourier spectrum and its compression independent of each other were mentioned. In the earliest studies, the effects of quantization of the Fourier spectrum of A/P were investigated. Direct quantization of both A/P was used in computer-generated holography for flat and 3D objects^49,50^. In the 2000 s, papers were published on the separation of complex holographic data^11,22,44–46^ into real/imaginary (R/I) parts instead of A/P parts. Because phase does not behave as amplitude (or intensity) under quantization, it is not so well suited for direct compression. However, separation on A/P and R/I parts was not compared for off-axis holograms earlier.
  The main objective of the most of papers devoted to compression by wavelets is assessment of the obtained compression ratio values, but not other parameters (for example, the value of losses of the compressed image quality). Application of the Gabor wavelet and the Fresnelet transform for compression is compared in^35^, but other wavelet types were not considered. Compression based on wavelet decomposition using additional wavelet coefficient coding was considered in^11^. Examples of reconstructed images using the Haar wavelet and uniform quantization have been demonstrated. A number of representations of object waves from inline digital holograms using the HEVC profile were analyzed in^20^. However, off-axis-type holograms and other variants of additional processing were not considered. Many popular methods of compression based on quantization, wavelets, and standard techniques of image compression are given in^12^, but comparisons and assessments of reconstruction quality are not given. Application of several different wavelets for compression of digital holograms was considered in detail in^51^; however, results of application of additional processing of decomposition coefficients are not given. Seven different wavelets were used for off-axis hologram compression based on coding of A/P of the filtered spectrum in^39^. However, use of the Fourier spectrum the R/I parts as a more preferable method of compression was not considered. In summary, in the majority of these papers, contrastive analyses of techniques of extra compression of wavelet coefficients (for example, various means of quantization) were not performed.
  Complex-valued holographic images (i.e., used in phase-shifting holography^52^) are usually given in articles focused on compression^12,17^. The corresponding object wave is often compressed (after preliminarily recording a number of phase-shifted digital holograms and filtering of twin and zero-order images)^20,22,51^. Only a few papers have addressed single digital hologram compression and only compression of the amplitude matrix has been considered^53^.
  For off-axis^54^ holograms, undesirable diffraction orders (zero-order and twin images) can be eliminated also^55^. Frequency filtering techniques are often used for reducing the informative holographic data size considerably^39,40^.
  Currently, off-axis hologram compression is researched for problems in microscopy (images of red blood cells^14^, micro bio objects^15^, etc.), terahertz systems^56^, 3D diffuse grayscale^57^ and color^58^ objects coding and imaging, computer-generated depth images representation^59^, next generation 3DTV applications^35,60^, quick volume scene displaying^61^, interferometry^15^, data multiplexing^62^, etc. Compression of off-axis digital holograms by binarization or direct quantization is used for visualization of optical scanning holographic data^63,64^, digital micromirror (DMD) applications^29,64^, fast printing and watermarking^65^, etc.
  There are two main aims of the paper:
- to compress single off-axis digital holograms using separation of the filtered spectrum into R/I parts and
- to perform a comparative analysis of the obtained results with those for compression by separation of the spectrum into A/P parts.
  Hologram compression based on a combination of a number of methods is analyzed, specifically,
- hologram frequency filtering,
- separation of the obtained spectrum into components,
- the wavelet transform, and
- additional processing by quantization and thresholding.

## Methods

### Wavelet compression of standard images and digital holograms

**Figure 1:**
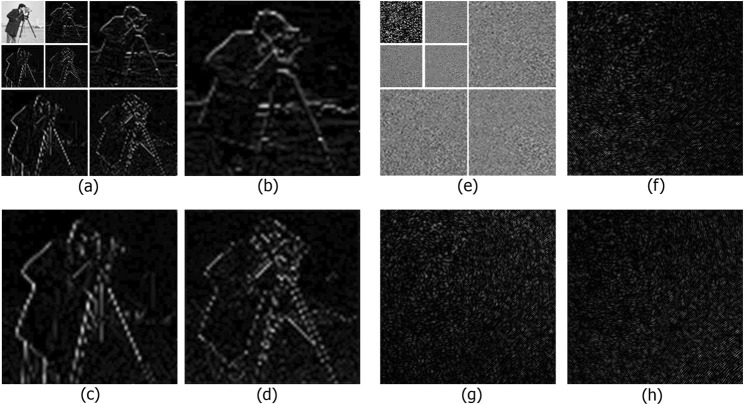
Wavelet transform coefficients of the image and of the synthesized digital hologram: horizontal (**b**,**f**); vertical (**c**,**g**); diagonal (**d**,**h**); the same ones for the three-level wavelet decomposition (**a**,**e**).

The wavelet transform^66^ of an array *x*(*t*) comprising the wavelet function *ψ*(*t*) can be defined in the following way:

1 $${T}_{m,n}={\int }_{-\infty }^{\infty }x(t){\psi }_{m,n}^{\ast }(t)dt,$$

where *T*_*m*,*n*_ are wavelet coefficients. There are different types of wavelet transforms^67,68^: orthogonal, semi-orthogonal, biorthogonal, symmetric, asymmetric, etc. Commonly, they are defined by a wavelet function and a scaling (mother) function, though these functions in their explicit forms do not participate in the decomposition and reconstruction algorithms. The ability of filters to use the available redundancy of signals is bound to properties of these functions. Limits at “the infinite iteration” of filters are defined by these functions. Smooth functions are approximated by shifts of the scaling function better than rough ones. As a result, the smooth signal is better compressed. Wavelets with the same wavelet and scaling functions can differ by the number of vanishing moments, *N*. *N* defines the degree of polynomials, which are removed by decomposition on the constructed wavelet basis:

2 $$\int {t}^{2}\psi (t)dt=0\,(l=0,1,\ldots ,N-1),$$

where *ψ*(*t*) is an orthogonal wavelet. Examples of the application of wavelet decomposition to natural photoimages and to fragments of hologram are given in Fig. 1. The result of wavelet decomposition^69^ is represented as a set of approximating (*A*), horizontal (*H*), vertical (*V*), and diagonal (*D*) coefficients of the transform. The basic distinguishable elements of the image are contained in the approximating coefficients while intermediate values of the initial image are in other ones. These coefficients are detailed and scaled.

In the case of compression of natural images, wavelets allow one to achieve high compression ratios with insignificant defects of the compressed images^67,69^. Gradient transitions of brightness values of standard photoimages can be described by a small quantity of coefficients. Respectively, in that case, the image with smooth transitions of brightness can be considerably compressed by wavelet decomposition. There are usually areas with gradient transitions in standard photoimages. At the same time, holograms are interference patterns and contain small elements with sharp differences of brightness in adjacent pixels. Consequently, decomposition of photoimages is more effective than decomposition of holograms and provides high compression ratios (of 20–50^70–72^ and up to 100^70,71,73^) with minimal loss of information or even without quality loss. These methods give lower compression ratios for the holograms. As a result, extra thresholding, quantization of wavelet coefficients, and subsequent processing by lossless compression (for example, entropy coding^74^) can be applied for increasing the hologram compression ratio.

In this paper, Daubechies^67,68^, Meyer^67,68^, symlet^68^, coiflet^68^, biorthogonal^69^, and reverse biorthogonal wavelets^69^ with different numbers of vanishing moments are used. Extra compression techniques (threshold processing and quantization of wavelet coefficients) are applied.

### Wavelet coefficient thresholding

Threshold processing (zeroing on a threshold) is a popular compression technique for wavelet decomposition coefficients^69^. The most popular algorithms are hard and soft thresholding. In hard thresholding, values less than a determined meaning of the threshold are reset to zero:

3 $$D(n,m)=\{\begin{array}{l}D(n,m),\,{\rm{if}}|D(n,m)| > \tau ,\\ 0,\,{\rm{if}}|D(n,m)|\le \tau ,\end{array}$$

where *D*(*n*,*m*) denote detailing coefficients, (*n*,*m*) are the coordinates of the detailing coefficients, *τ* is the threshold value, and |…| indicates the modulus of the value. Threshold processing of coefficients allows one to increase the compression ratio; however, the quality of the image is reduced.

### Wavelet coefficient quantization

Quantization is another method of additional wavelet coefficient compression^69^. In quantization, the large quantity of wavelet coefficients is decreased to a number of meanings (quantity of quantization levels). There are various quantization methods^12,13,26,27^. Most can be divided into several groups: uniform or nonuniform, direct or indirect, iterative or noniterative, scalar or vector, etc. Uniform quantization is based on uniform fragmentation of the dynamic range of the initial image into parts with equal length of elements^10,12^. Nonuniform methods are usually more qualitative than uniform ones because they address specifications of nonuniform histogram representations of compressed images^25–27^. Direct quantization is one of the most popular, easiest, and fastest methods of compression and allows one to achieve quick and visually essential compression of images^21–27^. Indirect quantization is not separated from entropy coding and is gaining popularity nowadays^52^. Iterative methods of quantization are based on repetition of a considerable number of iterations at the expense of what quality of image compression is achieved; however, the speed of image processing considerably decreases as a function of image quality. An example of iterative quantization is the clustering method based on self-training cycles in neural networks^75^. Noniterative methods operate during only one iteration. As a result, the quality of reconstruction is poorer but the processing speed is increased considerably. The most popular and fastest noniterative methods are methods of scalar quantization. The majority of quantization resource-intensive iterative methods process one file during a considerable time (tens and hundreds of seconds) with MATLAB using a standard computer^39^. However, the simplest methods of scalar quantization process a hologram during fractions of a millisecond or of a second.

### Combined digital hologram compression algorithms

The previously^39^ investigated combined algorithm for hologram compression included the following steps:

- frequency filtering of unwanted diffraction orders (twin and zero-order images),
- obtaining an array with A/P parts of the Fourier spectrum of the filtered hologram,
- performing wavelet decomposition of the separated A/P parts,
- threshold processing of the wavelet coefficients of A/P parts, and
- quantizing the thresholded wavelet coefficients of A/P parts.
  For increasing compression ratios, lossless techniques can be applied for further processing of the quantized thresholded wavelet coefficients.
  Use of the R/I parts of the Fourier spectrum instead of A/P should provide higher reconstruction quality. The combined algorithm does the following:
- frequency filtering of the twin and zero-order images,
- separating the R/I parts of the Fourier spectrum of the filtered hologram,
- performing wavelet decomposition of the separated R/I parts,
- threshold processing of the wavelet coefficients of both components, and
- quantizing the thresholded wavelet coefficients of both components.

Diffraction orders are spatially separated in the spectral plane of an off-axis hologram. The informative object order occupies only part of this spectrum. Therefore, use of pixels with a useful diffraction order only allows one to minimize the saved information size. The compression ratio in this case can be calculated as the ratio of the size of the informative order to the total size of the hologram Fourier spectrum.

Besides compression, frequency filtering allows one to improve the quality of reconstruction if it is intersected with zero-order or twin images. In the literature, various methods of filtering of undesirable diffraction orders are given. The most widespread method is truncation of the area of spatial frequencies^55^. In this case, all elements of the hologram’s Fourier spectrum except the part with object diffraction are nullified. This method gives good results in terms of reconstructed image quality.

**Figure 2:**
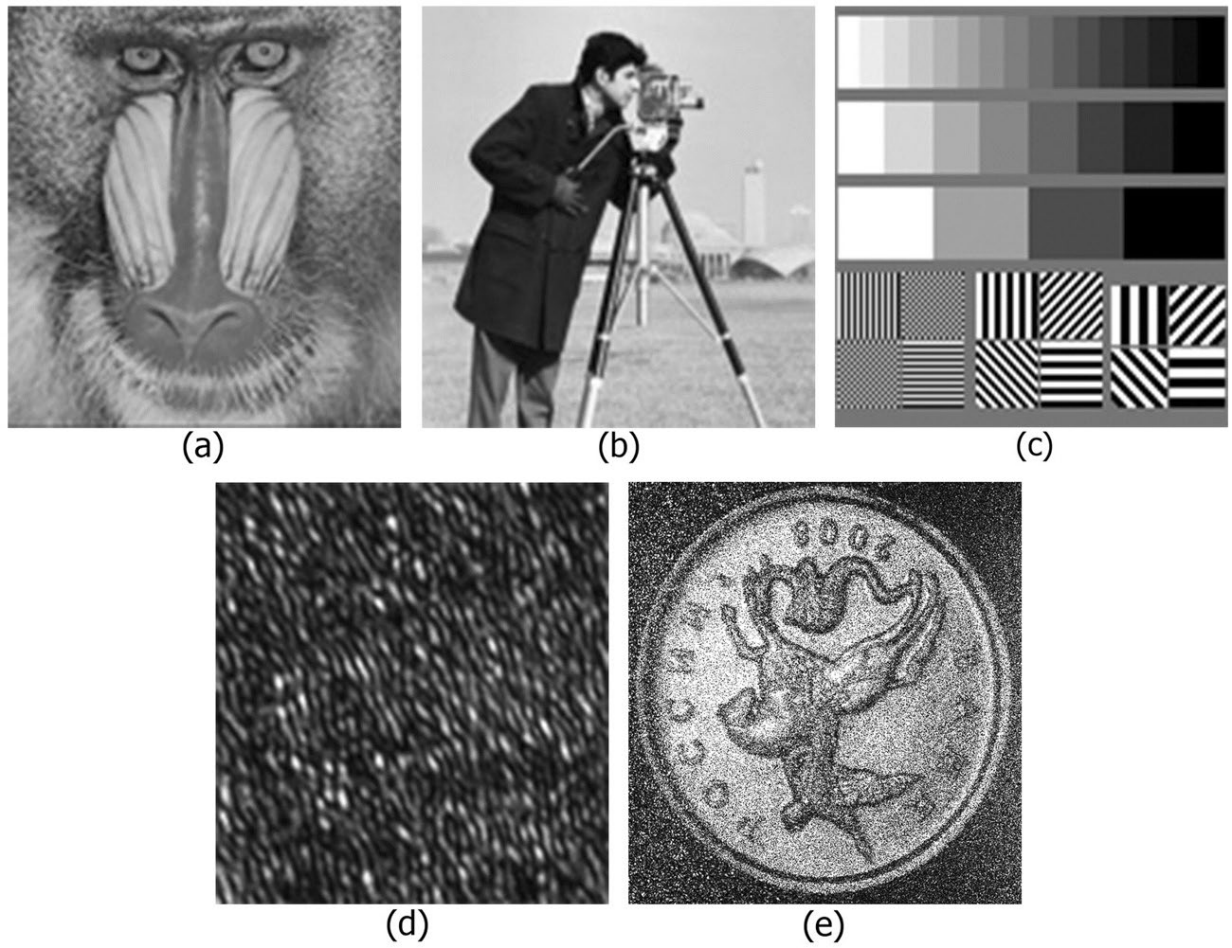
Test grayscale images (**a**–**c**); a fragment (128 × 128 pixels) of an optically recorded hologram (**d**); and a reconstructed object image (**e**).

Experimentally recorded digital holograms and synthesized holograms of 3D scenes with a number of binary and grayscale flat objects with up to 2048 × 2048 pixels are considered in this paper. Examples of images for hologram synthesis are given in Fig. 2. The standard images have 256 (Fig. 2(a,b)) and 16 (Fig. 2(c)) gradations of brightness. A fragment (128 × 128 pixels) of an optically recorded hologram with a size of 2048 × 2048 pixels and the reconstructed image are shown in Fig. 2(d,e)^40,76^, respectively. A synthesized hologram of a grayscale flat object shown in Fig. 2(a) and an optically recorded hologram of a 3D object shown in Fig. 2(e) will be used for numerical experiments. The illumination wavelength was 532 nm. The hologram pixel size was 9 × 9 *μ*m. The distance between different objects and the hologram plane was ranged from 0.4 m to 1.5 m. Object binary or grayscale images were positioned in the left corner of the full object field. The object phase was randomly distributed from 0 to 2*π*. Therefore, the radiation after propagation through the object or reflection from the object is diffuse radiation. The distribution of the object wave in the hologram plane was calculated by using the Fresnel diffraction method^4^:

4 $$O(u,v,z)=\exp \{ikz\}/(i\lambda z)\exp \{ik({u}^{2}+{v}^{2})/(2z)\}FFT\{{O}_{0}({x}_{0},{y}_{0},0)\exp \{[ik({x}_{0}^{2}+{y}_{0}^{2})/(2z)]\}\},$$

where *FFT*{…} is the fast Fourier transform, *k* is the wave number, *z* is the distance from the object to the hologram, (*x*_0_, *y*_0_, 0) are the coordinates in the object plane, (*u*, *v*, *z*) are the coordinates in the hologram plane, and *O*_0_(*x*_0_, *y*_0_, 0) is the transmission of the initial object. The hologram was synthesized by adding a normally falling plane reference wave *R* to the obtained object wave:

5 $$H(u,v)=|O(u,v,z)+R{|}^{2}=|O(u,v,z)+\sqrt{p\langle |O(u,v,z){|}^{2}\rangle }{|}^{2},$$

where *R* is the amplitude of a flat reference wave, *p* is the ratio of the average intensity of the reference wave to that of the object wave, 〈…〉 is the average value, and |…| indicates the modulus. Images from the hologram were reconstructed by using the Fresnel diffraction method (see Eq. 4).

The peak signal to noise ratio (PSNR) value^77^ was used as a measure of reconstructed image quality. A more detailed description of the first step of the algorithm (frequency elimination of unwanted diffraction orders for off-axis digital holograms) with examples is presented in^39^. In the underlying experiments, 88–90% of the square of the Fourier spectrum of synthesized holograms (with initial size of 1024 × 1024 pixels) and 83% of the Fourier spectrum of optically recorded holograms (with initial size of 2048 × 2048 pixels) were eliminated.

## Results

### Thresholding of wavelet coefficients of A/P and R/I parts

**Figure 3:**
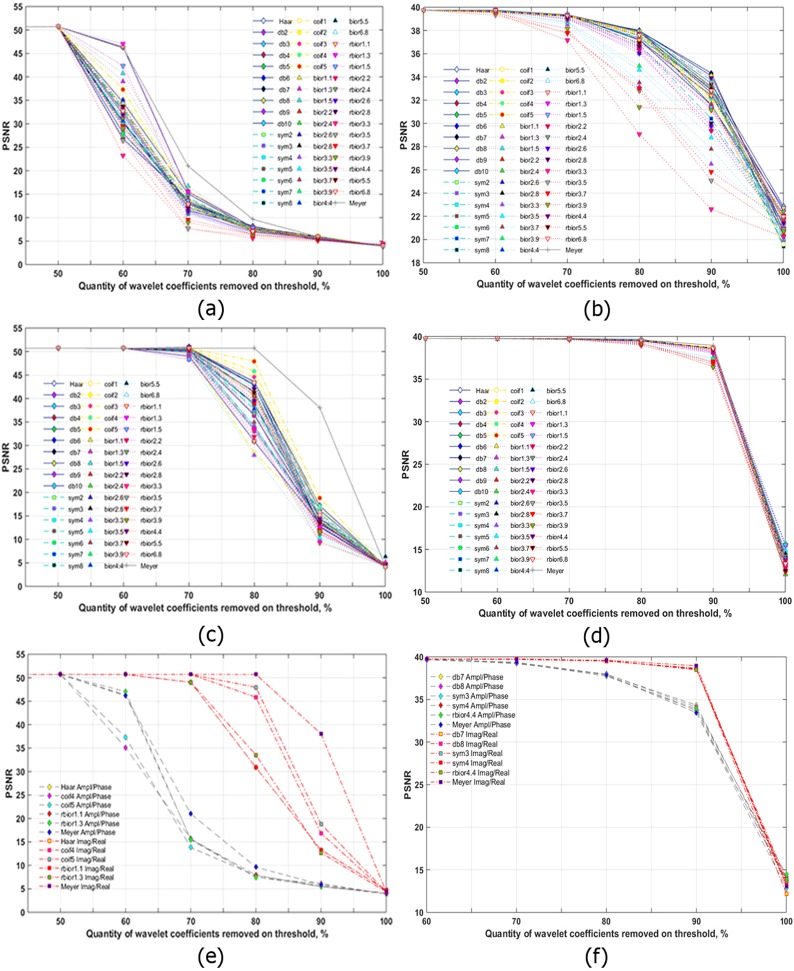
Quality of the reconstructed object from the synthesized hologram (**a**,**c**,**e**) and the experimentally registered hologram (**b**,**d**,**f**) versus the quantity of wavelet coefficients removed by the threshold for a three-level wavelet decomposition. A/P parts of the filtered spectrum are compressed (**a**,**b**). R/I parts of the filtered spectrum are compressed (**c**,**d**). The best six wavelets are shown for both cases in (**e**,**f**).

Threshold processing of wavelet decomposition coefficients was applied to compress separated A/P and R/I parts of the Fourier spectrum of holograms after frequency filtering. 51 wavelets were used for hologram compression in this paper. Similar dependencies for the case of separated A/P parts of hologram spectra were considered for 7 wavelets in^39^. The dependencies of PSNR versus the percentage of wavelet coefficients nullified by the threshold (quantity of zeros in the sets of wavelet decomposition coefficients) were obtained, as shown in Fig. 3(a,c). The cases of three-level wavelet decomposition of the A/P and R/I parts of a synthesized hologram are given.

In the case of compression of A/P parts during removal of no more than 50% of the coefficients, the quality of the image does not change and is poorly differs for all wavelets. As more coefficients are removed, there is a more noticeable distinctions between the values of quality metrics for the different wavelets. The highest PSNR was obtained for the Haar, reverse biorthogonal 1.1 and 1.3, biorthogonal 1.3, and Meyer wavelets. In terms of compression ratio and quality of the reconstructed images, threshold zeroing with ~60–70% of the wavelet coefficients is optimum. In the case of compression of R/I parts, the most optimum thresholding for all wavelets is ~80%. Values of PSNR are not changed during removal of 70% of the wavelet coefficients for compressed R/I parts. The highest PSNR at removal of >70% of the coefficients was obtained using the Meyer wavelet and coiflets 3, 4, and 5. For a more suitable visual comparison, wavelets with the highest quality reconstruction are shown in Fig. 3(e) for both cases. The average PSNR for R/I parts for 90% nullified coefficients is 11.8 dB (or a factor of 2.69) higher than that for A/P parts.

Figure 3(b,d,f) show similar dependencies for experimentally recorded holograms for the three-level wavelet decomposition. For A/P parts, the PSNR begins to decrease during removal of 60% of the wavelet coefficients. In this case, the highest PSNR was obtained using Daubechies 7 and 8, symlet 4, Meyer, and reverse biorthogonal 4.4 wavelets. The most optimum quantity of decomposition coefficients truncated by wavelet thresholding is in the range of 70–80%. In the case of R/I compression, the most optimum thresholding is higher than the that for A/P compression and is ~80–90%. PSNR values almost do not change during removal of 70% of the wavelet coefficients. The highest value of PSNR was obtained using Meyer wavelets, coiflets, symlets, and Daubechies wavelets. The PSNR for R/I parts for 90% nullified coefficients is 4.8 dB (or 14.3%) higher than that for A/P parts. In Fig. 3(f), both cases are demonstrated. The most applicable wavelets for both cases are used. The various methods yield almost identical results for R/I compression. The difference between PSNRs is >1 dB. Among A/P compression methods, the results are similar to each other. PSNRs differed by up to ~1 dB.

In conclusion, use of R/I parts allows up to a twofold increase of compression ratios in comparison with what A/P parts provide. A/P compression with 60–70% of the wavelet coefficients removed by the threshold gives almost the same reconstruction quality as 80% removal in the R/I case. Removing 90% of the wavelet coefficients while maintaining a relatively high quality of reconstruction is possible using R/I parts. This threshold level can be considered as the most optimum value in terms of compression ratio and reconstruction quality.

### Uniform quantization of wavelet coefficients of A/P and R/I parts

After separation of A/P or R/I parts of the spectrum, the wavelet transform is applied. Further wavelet coefficients are quantized. First, uniform quantization by level is analyzed as the most popular method and one of the fastest and simplest ones.

**Figure 4:**
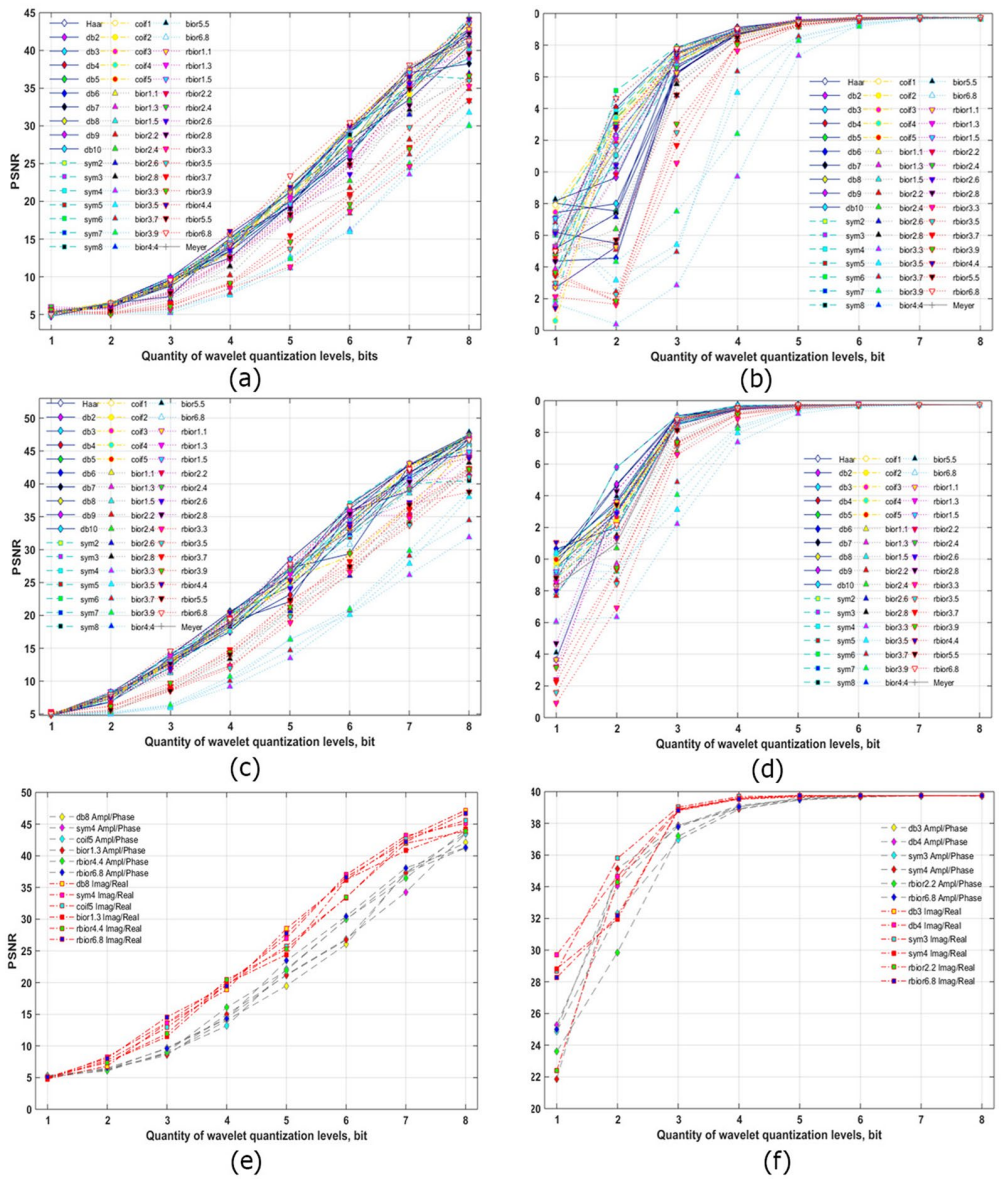
Quality of the reconstructed object from a synthesized hologram versus the number of wavelet coefficient levels for two-level wavelet decomposition (**a**,**c**.**e**) and from the experimentally registered hologram versus the number of wavelet coefficients for three-level wavelet decomposition (**b**,**d**,**f**). Wavelet coefficients were uniformly quantized. A/P parts of the filtered spectrum are compressed (**a**,**b**). R/I parts of the filtered spectrum are compressed (**c**,**d**). The best six wavelets are shown for both cases in (**e**,**f**).

The obtained dependencies of PSNR versus number of gradations of quantized wavelet decomposition coefficients of A/P and R/I parts of the filtered hologram spectrum are shown in Fig. 4(a,c,e). Components were compressed by two-level wavelet decomposition. Threshold processing was not used. Decreasing the number of quantization bits causes the PSNR to almost linearly decrease. The highest PSNRs in the A/P case were obtained using reverse biorthogonal 6.8, biorthogonal 6.8, reverse biorthogonal 4.4, coiflets 5 and 1, and Daubechies 9 wavelets. In case of less than 3-bit quantization, many informative wavelet coefficients are lost, so, on the reconstructed images, the initial object is almost indistinguishable. However, in case of R/I quantization, PSNRs almost linearly decrease up to 1 bit (see Fig. 4(e)). The highest values of PSNR are achieved using reverse biorthogonal 6.8, biorthogonal 1.3, symlet 4, and Daubechies’s 8 wavelets. The average difference between PSNRs for A/P and R/I parts is ~3.8 dB (i.e., R/I provides 18.6% higher SNR than does A/P) in Fig. 4(e). The maximum difference in dB is for 6 bits and is 7.1 dB (or 25.2%). The maximum difference in percent is for 3 bits and is 4.0 dB (or 43.6%). Therefore, R/I parts can provide better quality by up to a factor of 1.4 for the same value of coefficient quantization.

The similarly obtained dependencies for the experimentally recorded hologram are shown in Fig. 4(b,d,f). In the case of A/P compression, the highest PSNRs were obtained using symlet 6, Daubechies 4, reverse biorthogonal 6.8, and biorthogonal 4.4 wavelets. For compression of the experimentally registered hologram R/I parts, the best results were achieved using symlet 3, Daubechies 3, 4, and 9, and reverse biorthogonal 2.2 and 2.8 wavelets. The best results for both types of components are shown in Fig. 4(f). The highest quality of reconstruction is achieved with >3 bits for R/I and >4 bits for A/P parts. The average PSNRs are comparable. However, results for R/I parts of the filtered hologram spectrum are a bit better than those for A/P parts, especially for the 1-bit case. In that case, the average PSNR is higher by 3.5 dB (or 14.5%) when compared with A/P compression. With consistent application of both quantization and threshold processing, the difference between these two cases will become more significant.

### Thresholding and quantization of wavelet coefficients of A/P and R/I parts

**Figure 5:**
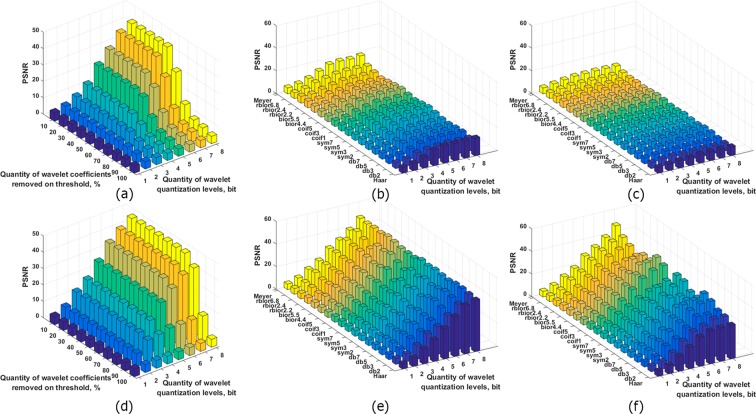
Quality of the reconstructed object from the synthesized hologram versus the number of wavelet coefficient gradations and the quantity of wavelet coefficients removed by the threshold for the two-level reverse biorthogonal 6.8 wavelet decomposition (**a**,**d**) and for two-level different wavelets decomposition (**b**,**c**,**e**,**f**). 70% (**b**,**e**) and 80% (**c**,**f**) coefficients are removed by the threshold. A/P (**a**–**c**) and R/I parts (**d**–**f**) of the filtered spectrum are compressed.

After hologram frequency filtering, separation of spectral parts, and applying the wavelet transform, the wavelet coefficients were threshold processed and quantized in combination. The dependencies of PSNR versus the number of gradations of quantized wavelet coefficients and the quantity of wavelet coefficients nullified by the threshold are shown in Fig. 5(a,d). Cases of A/P (a) and R/I (d) of the filtered synthesized hologram spectrum of a grayscale image for two-level decomposition by a reverse biorthogonal 6.8 wavelet are considered. High PSNRs are achieved in the A/P case with no more than 50% nullified coefficients at 7 and 8 bits. The highest results for R/I are achieved in case of threshold removal of ~70–80% of the coefficients at 7- and 8-bit quantization of wavelet coefficients. The average difference between the best results for R/I and A/P is 6.3 dB (or 15.4%).

Figure 5(b,c,e,f) show PSNRs in cases of the 18 best wavelets for compression of A/P (Fig. 5(b,c)) and R/I (Fig. 5(e,f)) for synthesized holograms. Both cases are considered for 70% (Fig. 5(b,e)) and 80% (Fig. 5(c,f)) coefficient removal by the threshold and 1- to 8-bit quantization. In these figures, PSNRs for R/I compression are much higher than those for A/P compression. The average difference between the highest PSNRs is ~33.2 dB (or a factor of 3.36) for threshold removal of 70% of the coefficients and ~35.8 dB (or a factor of 6.11) for threshold removal of 80% of the coefficients. Compression of R/I by removing 70% of the wavelet decomposition coefficients and its quantization for a high number of gradations (7 or 8 bits) for all wavelets provide almost identical results. However, increasing the quantity of nullified threshold coefficients (in case of 80% removed coefficients, see Fig. 5(c,f)), leads to an increase in the difference between PSNRs for different wavelet transforms. The highest PSNRs are achieved in cases of Meyer and reverse biorthogonal 6.8 wavelets, coiflets 3 and 5, symlets 5 and 7, and Daubechies 5 and 7 wavelets.

**Figure 6:**
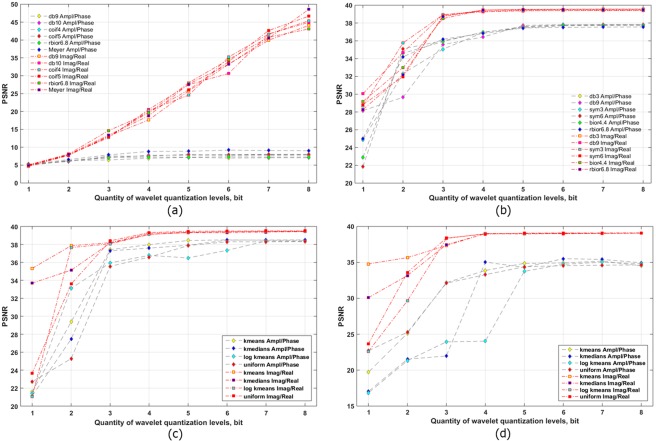
Quality of the reconstructed object from the synthesized hologram for two-level wavelet decomposition (**a**) and from the experimentally registered hologram for three-level wavelet decomposition (**b**–**d**) versus the number of wavelet coefficient levels. 80% (**a**,**b**), 75% (**c**), and 85% (**d**) coefficients are removed by the threshold. A/P and R/I parts of the filtered hologram spectrum are compressed. The best six wavelets in case of uniform quantization are shown in (**a**,**b**), and different quantization methods are shown in (**c**,**d**).

The obtained dependencies of PSNRs on the number of quantized wavelet coefficient gradations for both types of components for synthesized and optically recorded holograms are shown in Fig. 6(a,b). Threshold zeroing of 80% of the coefficients at two-level and three-level wavelet decomposition was applied. In Fig. 6(a), the two types of dependencies differ significantly between PSNRs for A/P and R/I parts. The average difference in case of a high number of gradations (7 or 8 bits) is 35.5 dB (or a factor of 5.51). In case of 4–6 bits, the average difference is 18.7 dB (a factor of 2.39).

In the case of optically recorded hologram compression, the difference is not as significant (see Fig. 6(b)). For 3–8 bits, the quantization difference between PSNRs for A/P and R/I cases is only 2.1 dB (or 5.7%). However, with decreasing number of gradations, the difference between PSNRs begins to increase. For 1 bit, it is equal to 4.3 dB (or 17.6%).

For a more detailed investigation of wavelet coefficient compression, iterative and noniterative methods of quantization were applied. The following iterative methods of quantization were used: dynamic kernels (k-means)^78^, dynamic kernels with a logarithmic transform, and k-medians^78^. Noniterative methods (uniform quantization by level)^46^ were also considered. The results are shown in Fig. 6(c,d). The Meyer wavelet, as one of the best methods in terms of reconstruction quality and compression ratio, was used for A/P and R/I parts of an optically recorded hologram. Cases of 75% and 85% nullified coefficients were used. For a high number of gradations (>5 bits), the results for A/P quantization and R/I compression are similar. With decreasing number of gradations, the highest PSNRs are achieved using iterative vector methods (k-medians and k-means). For 85% nullified elements, the average PSNR for A/P is decreased by 4 dB (or 13.3%) while that for R/I decreased only by 0.7 dB (or 2.0%). As a result, movement to 90–95% leads to an increasing advantage of R/I based on the PSNR value. The average difference between A/P and R/I quantization with 75% thresholding is 2.8 dB (or 8.2%) to 7.2 dB (or 25.2%) for 2 bits. The average difference for 85% thresholding is 6.7 dB (or 22.2%) to 10.3 dB (or 37.5%) for 3 bits. Unlike the A/P case, there is no noticeable PSNR decrease for R/I compression at 1 or 2 bits.

If high-quality reconstruction of an object is more important than the compression ratio, then the optimum level of quantization can be considered as 4 or 5 bits. This result corresponds to previously published values in the literature^21,26,46^. However, in those papers, the hologram or object wave was directly quantized. In this paper, quantization is only one stage of compression. Optimal threshold removal of coefficients is ~90% for R/I and 70–80% for A/P. The compression ratio is *sim*80–100 for A/P in case of 4- or 5-bit quantization. However, compression of R/I allows one to decrease quantization up to 1 bit while obtaining a relatively high object quality. For this number of gradations, high compression ratios at relatively high reconstruction quality can be achieved. The obtained compression ratio is 380. Therefore, the optimum wavelet transform providing the least losses of quality can be defined according to wavelet decomposition levels, quantities of thresholded wavelet coefficients, and quantization gradations.

### Reconstructed images of 2D and 3D scenes from compressed holograms

**Figure 7:**
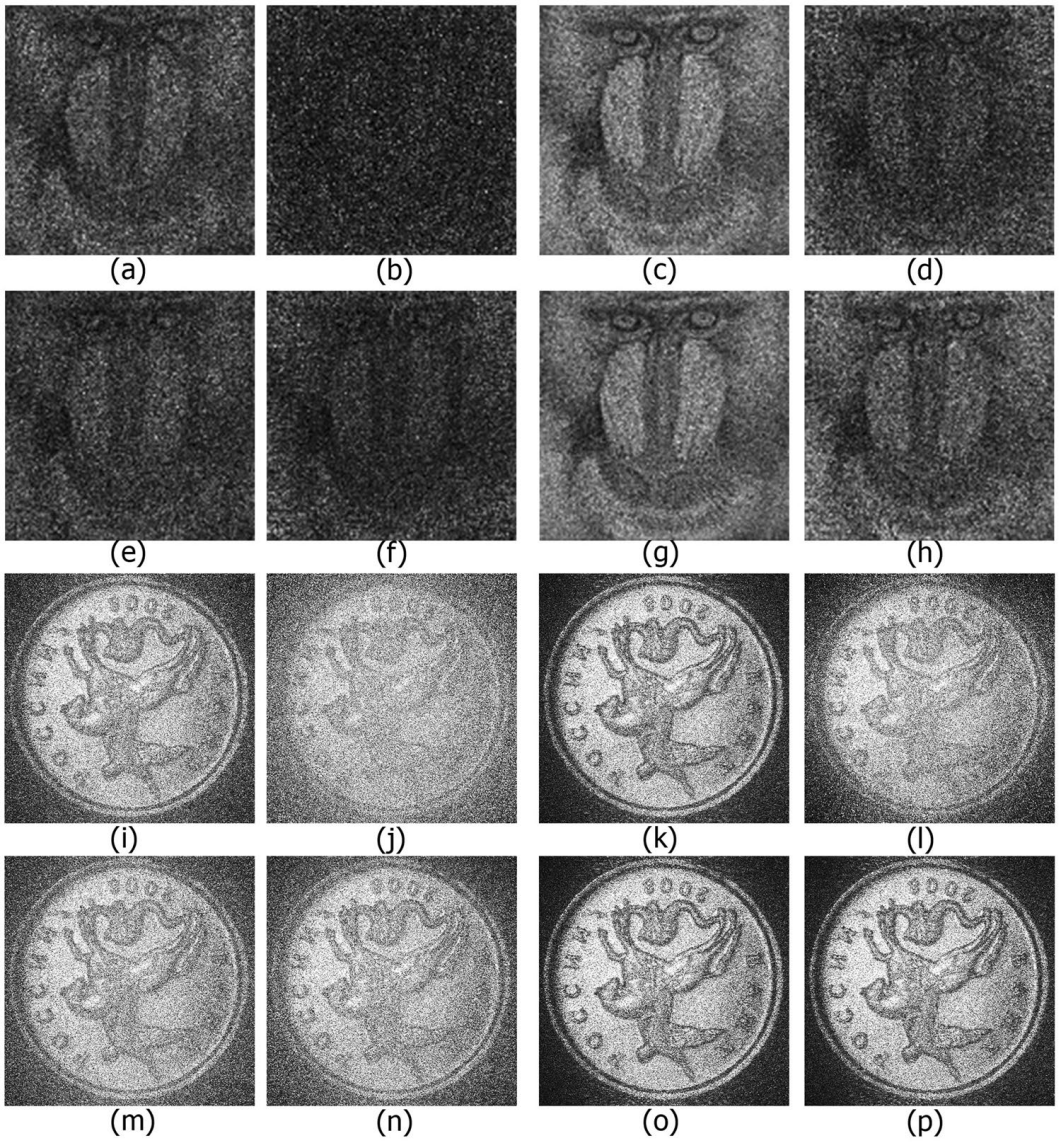
Reconstructed object from synthesized holograms with 3 (**a**–**d**) and 4 (**e**–**h**) bits and from experimentally registered holograms with 2 (**i**–**l**) and 3 (**m**–**p**) bits of reverse biorthogonal 6.8 wavelet transformed A/P parts (**a**,**b**,**e**,**f**,**i**,**j**,**m**,**n**) and R/I parts (**c**,**d**,**g**,**h**,**k**,**l**,**o**,**p**) of the filtered hologram spectrum. 70% (**a**–**d**,**i**–**l**) and 80% (**e**–**h**,**m**–**p**) coefficients are removed by the threshold. k-mean (**a**,**c**,**e**,**g**,**i**,**k**,**m**,**o**), k-median (**b**,**d**,**f**,**h**) and uniform (**j**,**l**,**n**,**p**) quantizations were used.

Figure 7(a–h) show reconstructed images from compressed synthesized holograms of a grayscale image for several implementations of additional compression of A/P and R/I wavelet decomposition coefficients of the filtered hologram spectrum. The reverse biorthogonal 6.8 wavelet transform, as one of the best in terms of reconstruction quality, was used with three-level decomposition. This level of decomposition can be determined as optimum because it provides the least loss of quality of the reconstructed images and the maximum compression ratio. Cases of 3- and 4-bit quantization and 70–80% removed coefficients are given. As expected, reconstructions from holograms with compressed R/I part have much higher quality than those with compressed A/P parts.

Figure 7(i–p) show the reconstructed images from optically recorded holograms. Quantization with 2 or 3 bits and threshold processing of 70% and 80% of wavelet decomposition coefficients are used. Iterative quantization using R/I parts gives the best quality of reconstruction in terms of both PSNR value and visual point of view. A/P parts require 4 bits to achieve the same quality that R/I parts need only 2 bits for (see Fig. 6(d)). The average difference between PSNRs for A/P and R/I component compression is 2.3 dB in case of 2 bits and threshold removal of 70% of the coefficients and is 7.3 dB in case of 2 bits and threshold removal of 70% of the coefficients.

**Figure 8:**
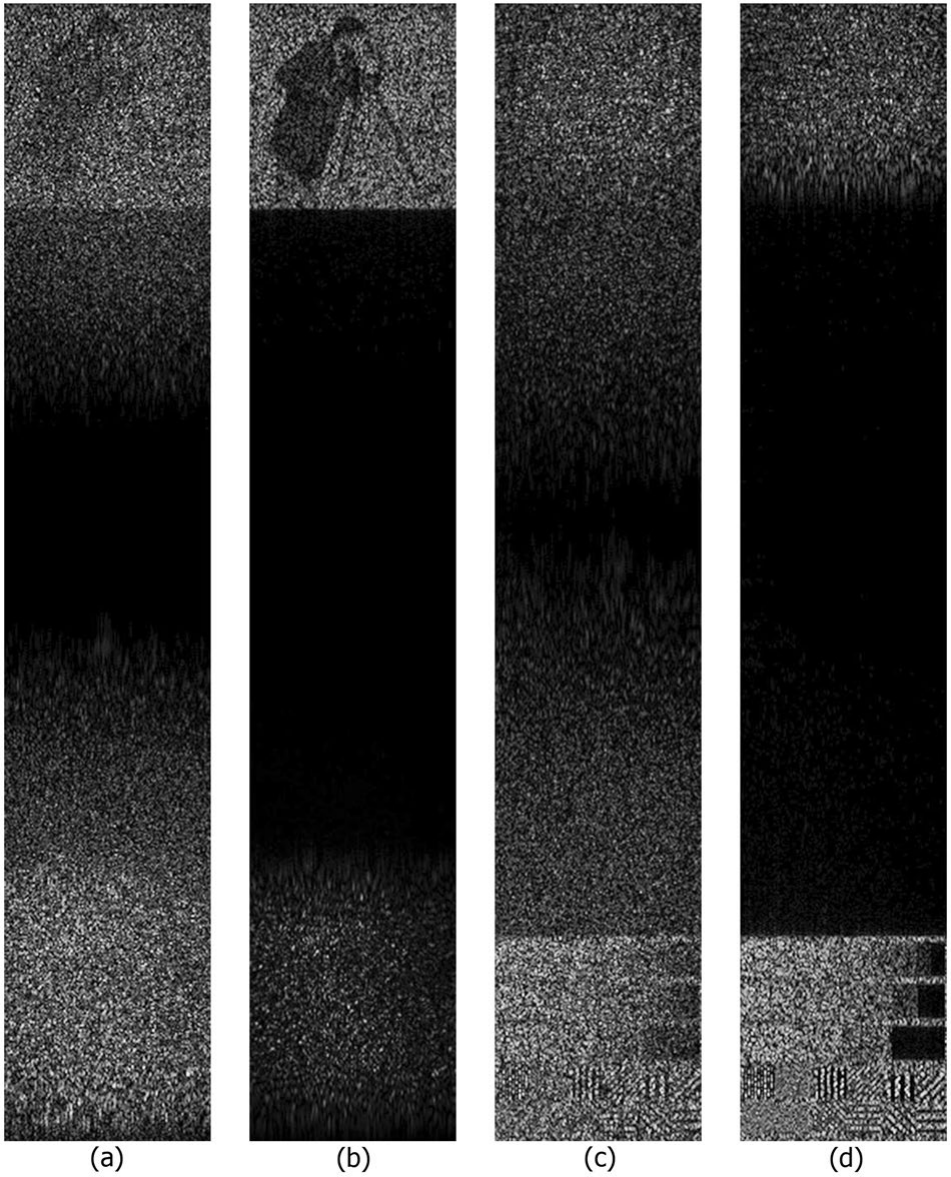
Reconstructed images from the synthesized holograms with 5 bits of reverse biorthogonal 6.8 wavelet transformed A/P parts (**a**,**c**) and R/I parts (**b**,**d**) of the filtered hologram spectrum. 90% coefficients are removed by the threshold. k-means were used for quantization. Images were reconstructed in planes of the first (**a**,**b**) and second objects (**c**,**d**).

Figure 8 shows the reconstructed images from synthesized holograms of a 3D scene. The scene consists of two 2D objects located in variant planes. The distances between the objects and the hologram are 0.62 and 1.09 m. 5-bit quantization and threshold truncation of 90% of the three-level wavelet decomposition coefficients are used. The highest quality of reconstruction is obtained using R/I parts and coefficient quantization of ≥4 bits. For higher compression ratios, fewer gradations should be used. The optimum level of quantization for high-quality object reconstruction is 5 or 6 bits.

The highest reconstruction quality of the grayscale image is observed in case of compressed R/I using >5-bit nonuniform quantization and 80% of the threshold nullified wavelet coefficients. The compression ratio in that case is ~70. In the case of compression of A/P for binary image quantization, up to 4 bits gives high reconstruction quality. This allows one to achieve compression ratios up to 80. However, compression of R/I parts allows one to increase the compression ratio by a factor of 2 when compared with A/P parts with the same quality of the reconstructed images (or to 160 overall).

In the case of Fig. 7(k), the compression ratio is 100. Further increasing the threshold value to 90% and decreasing of number of quantized wavelet coefficient gradations of the R/I parts of the spectrum to 2 yields a compression ratio of 380. Further increases in the compression ratio can be achieved through the use of lossless techniques^46^ for coding of the quantized thresholded coefficients of the wavelet transform.

## Conclusions

In this paper, off-axis synthesized and optically recorded digital holograms of up to 2048 × 2048 pixels of various objects were compressed by using a combination of methods consisting of hologram frequency filtering, separation of the Fourier spectrum of the filtered hologram into A/P or R/I parts, obtaining its wavelet decomposition by different transforms, and additional processing of wavelet coefficients. In total, 51 wavelets and four iterative and noniterative methods of quantization were used.

Processing of A/P and R/I parts was compared. As expected, R/I parts provide higher PSNRs because of the difficulties in directly quantizing the phase. The average difference between PSNRs is more than tens of percent (and up to hundreds of percent for several experiments) in favor of using R/I parts. In different experiments, various wavelets demonstrated better results. Meyer and reverse biorthogonal 6.8 wavelets, several coiflets, and some symlets can be considered as the most universal wavelets. The most suitable parameters and compression values and extra processing are defined. They provide high compression ratios and the reconstruction quality can be considered as average or high. A comparison of results allowed us to determine the most optimum compression parameters.

Use of A/P parts allows one to achieve compression ratios of up to 100 for average reconstruction quality and up to 190 for poor reconstruction quality (in the case of elimination of 90% of the spectrum, quantization on two gradations or 1 bit per pixel, and zeroing of 80% of the wavelet decomposition coefficients). Separation of the spectrum into R/I parts allows one to obtain a compression ratio of holographic information up to a factor of 380 (in the case of elimination of 90% of the spectrum, quantization on two gradations or 1 bit per pixel, and zeroing of 90% of the wavelet decomposition coefficients). In that case, object quality can be considered as average or high. For obtaining even better compression ratios, lossless algorithms can be additionally applied.

## Data Availability

The datasets generated during and/or analysed during the current study are available from the corresponding author on reasonable request.
